# To Study and Determine the Role of Anterior Segment Optical Coherence Tomography and Ultrasound Biomicroscopy in Corneal and Conjunctival Tumors

**DOI:** 10.1155/2016/1048760

**Published:** 2016-12-06

**Authors:** Katleen Janssens, Michelle Mertens, Noémie Lauwers, Rob J. W. de Keizer, Danny G. P. Mathysen, Veva De Groot

**Affiliations:** ^1^University of Antwerp, Antwerp, Belgium; ^2^Department of Ophthalmology, University Hospital Antwerp, Edegem, Belgium; ^3^Department of Ophthalmology, LUMC, Leiden, Netherlands

## Abstract

*Purpose*. To analyze and describe corneal and conjunctival tumor thickness and internal characteristics and extension in depth and size and shape measured by two noninvasive techniques, anterior segment optical coherence tomography (AS-OCT) and ultrasound biomicroscopy (UBM).* Design*. Systematic review.* Methods*. This systematic review is based on a comprehensive search of 4 databases (Medline, Embase, Web of Science, and Cochrane Library). Articles published between January 1, 1999, and December 31, 2015, were included. We searched for articles using the following search terms in various combinations: “optical coherence tomography”, “ultrasound biomicroscopy”, “corneal neoplasm”, “conjunctival neoplasm”, “eye”, “tumor” and “anterior segment tumors”. Inclusion criteria were as follows: UBM and/or AS-OCT was used; the study included corneal or conjunctival tumors; and the article was published in English, French, Dutch, or German.* Results*. There were 14 sources selected.* Discussion*. Several studies on the quality of AS-OCT and UBM show that these imaging techniques provide useful information about the internal features, extension, size, and shape of tumors. Yet there is no enough evidence on the advantages and disadvantages of UBM and AS-OCT in certain tumor types.* Conclusion*. More comparative studies are needed to investigate which imaging technique is most suitable for a certain tumor type.

## 1. Introduction

Since the early nineties ultrasound biomicroscopy (UBM) has been used for the imaging of the anterior eye segment and was soon discovered to be useful in the evaluation of superficial tumors. Anterior segment optical coherence tomography (AS-OCT) became available in the 21st century. One of the first series on the use of OCT in the evaluation of conjunctival tumors was published by Buchwald et al., in 2003 [1], followed by De Keizer and Razzaq in 2007 [2].

Corneal and conjunctival tumors can be visualized by AS-OCT and UBM, two noninvasive imaging techniques. Hereby, tumor thickness and internal characteristics and extension in depth and size and shape can be measured. Several small series have been published, evaluating the use and the quality of AS-OCT or UBM in assessing these tumors. It is still not clear which technique to use in certain tumor types. We therefore conducted a literature search in order to find an answer to the following question: how accurate are AS-OCT and UBM in determining tumor margins and tumor depth of conjunctival and corneal tumors and can they provide additional information guiding the diagnosis?

## 2. Material and Methods

### 2.1. Anterior Segment Optical Coherence Tomography

AS-OCT is an examination technique that uses reflected light waves in order to reconstruct a cross section of the examined tissue. Time-domain OCT measures the electromagnetic radiation in function of the time. This investigation can make up to 400 axial scans per second and has an axial resolution of 8–10 *μ*m. Spectral-domain OCT measures the wavelength of the reflected light and compares the image with a reference point by means of mirrors, which allows it to measure faster. This imaging technique also measures electromagnetic radiation and can make up to 25.000–50.000 scans per second. It has an axial resolution of 5–7 *μ*m. In comparison, spectral-domain OCT obtains more data in less time and with higher axial resolution [3, 4]. OCT of the anterior segment can achieve a 9-10 *μ*m axial and 15 *μ*m transverse resolution [5]. This enables it to visualize smaller details compared to those shown on ultrasound or MRI [6, 7]. Ultrahigh Resolution OCT (UHR-OCT) uses a similar technique to OCT but results in a higher resolution (3 *μ*m axial resolution) [8–10].

*Advantages and Disadvantages*. OCT has many important advantages. It is a widely available noncontact method without ionizing radiation. As a result, there are no risk of eye damage and no discomfort for the patient. OCT directly renders high resolution images while the examination takes less than 5 minutes and can be performed by an optometrist. In comparison with other competing imaging techniques, AS-OCT has a low penetration depth of 1–3 mm but offers a high axial resolution due to the use of short wavelength light (±830 nm mostly) [3, 5, 11]. AS-OCT is ideal for imaging structures from the surface of the eye to the level of the iris [5]. A disadvantage of AS-OCT is that it cannot visualize structures behind pigmented lesions, like the iris, and cannot visualize early pathological changes smaller than 5 *μ*m such as early dysplasia [5, 7].

### 2.2. Ultrasound Biomicroscopy

UBM is an examination technique that uses sound waves to analyze the structures as in the classic ultrasound investigation. However, in UBM a higher frequency is used which consequently allows more detail but less penetration into the tissue [12]. For the anterior segment, transducers are used with a frequency of 35–50 MHz. UBM has an axial resolution of 42 *μ*m and a tissue penetration of 4-5 mm [5, 13].

*Advantages and Disadvantages*. The most important advantage of UBM is that it can penetrate opaque tissue [14]. In contrast, AS-OCT is unable to do this. UBM also has a broad field of view [15]. UBM is ideal for imaging structures from the surface of the eye to the anterior vitreous [5]. Disadvantages are the limited penetration depth of 4-5 mm and the limited depth resolution. UBM requires topical anesthesia and the application of an eyecup filled with fluid in contact with the eye surface causing mild discomfort for the patient. Unfortunately the accessibility to UBM is limited to the larger centers.

### 2.3. Methods

We performed a specific literature search of peer reviewed published journal articles in the following stages.

*Stage 1.* Comprehensive search of 4 databases (Medline, Embase, Web of Science, and Cochrane Library). Articles published between January 1, 1999, and December 31, 2015, were included. We searched for articles using the following search terms in various combinations: “optical coherence tomography”, “ultrasound biomicroscopy”, “corneal neoplasm”, “conjunctival neoplasm”, “eye”, “tumor”, and “anterior segment tumors”. 

*Stage 2.* Articles were first selected based on title and subsequently on abstract and full text. Inclusion criteria were as follows: UBM and/or AS-OCT was used; the study included corneal or conjunctival tumors; the article was published in English, French, Dutch, or German. We could include 14 articles.

**Figure 1:**
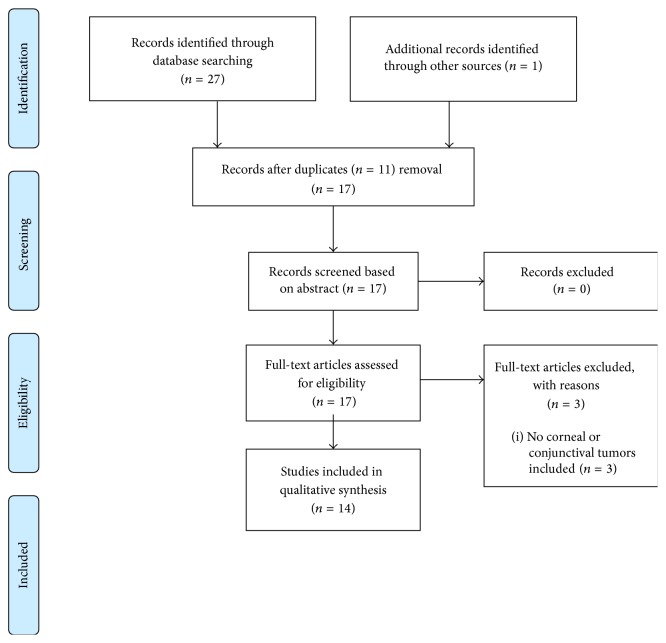
Flow diagram of literature search.

The flow diagram of the literature search is shown in Figure 1.

## 3. Results

We analyzed 14 papers: 6 studies on UBM, 6 on OCT, and 2 studies that compared the two imaging techniques.

All studies are listed in Tables 1, 2, and 3.

### 3.1. UBM

Lanzl et al. [16], Grant and Azar [17], and Hoops et al. [18] all studied the use of UBM in limbal dermoids, respectively, on 2, 1, and 8 patients. They all found a hyperreflective lesion compared to the cornea. They concluded that UBM is a useful preoperative tool in limbal dermoids [16, 17] but insufficient in some cases to measure corneal penetration [17].

Buchwald et al. demonstrated in 28 patients with solid tumors of the conjunctiva that UBM can be an additional diagnostic tool, for example, to determine the margins of the solid tumors or cysts [19].

Similarly to the study of Buchwald et al., Lin et al. proved in 2 cases that UBM is a useful tool to show cysts in conjunctival lesions. This technique could be used for delineating the extent of the lesion before excision [20].

Ho et al. studied the assessment of tumor thickness in three conjunctival melanomas by means of UBM. They concluded that high frequency UBM is useful for estimating tumor thickness in conjunctival melanomas and can be used to determine the tumor depth prior to surgical resection [21].

### 3.2. AS-OCT

The study of Shields et al. on 22 conjunctival nevi demonstrated that all margins of conjunctival nevi, including the deep borders, could be visualized by AS-OCT. AS-OCT images showed a high resolution in 100% of anterior borders and 82% of posterior borders [22]. Some of the images were affected by deep optical shadowing, especially in pigmented nevi (86%). The sensitivity of AS-OCT for the detection of intrinsic cysts in a conjunctival nevus is 80%, the specificity is 100%, the positive predictive value is 100%, and the negative predictive value is 60%. Thus, AS-OCT ensures high resolution images of conjunctival nevi, it can delineate the borders of the lesion, and it can demonstrate the presence of intralesional cysts [22]. The main drawback of OCT is the presence of optical shadowing in pigmented nevi. The thickness of nevi in the study ranged from 0.1 mm to 1.7 mm measured with AS-OCT [22].

Welch et al. studied the difference between the measurements of a pterygium by slit-lamp examination and by AS-OCT imaging [23]. When measuring the distance from the apex of the pterygium to the limbus in 13 eyes, AS-OCT gave significantly better reproducible results. Therefore, they concluded that AS-OCT allows us to accurately determine the extension of a pterygium on the cornea [23].

Shousha et al. studied the use of UHR-OCT in the diagnosis and follow-up of conjunctival and corneal intraepithelial neoplasia (CCIN). UHR-OCT images of the 7 lesions discerned a thickened hyperreflective epithelium and abrupt transition from normal to hyperreflective epithelium. Their results demonstrated that macroscopically resolved residual tumor nodules can be visualized by UHR-OCT. They concluded that UHR-OCT is useful for guiding diagnosis and treatment follow-up of CCIN [9].

The results of the study of Kieval et al. showed that UHR-OCT of the anterior segment could be an accurate tool in differentiating ocular squamous cell carcinoma from pterygium [8]. The difference in measured epithelial thickness allows ophthalmologists to make a distinction. The average epithelial thickness in the 17 epithelial squamous cell carcinomas (SCC) was 346 *μ*m, compared to 101 *μ*m in the 17 pterygia. Using a cut-off value of 142 *μ*m results in a sensitivity of 94% and a specificity of 100% [8] in differentiating SCC from pterygia.

In another study of Shousha et al., the use of UHR-OCT in the diagnosis of 54 ocular surface lesions was studied. It was demonstrated that when the clinical diagnosis of ocular surface lesions was uncertain, UHR-OCT images provided optical signs indicating more specific diagnosis and management. They concluded that this imaging technique can visualize the structure and location of the lesion and as such can aid in guiding the diagnosis and management [10].

Nanji et al. studied the use of high resolution, spectral-domain optical coherence tomography (HR-OCT) in the diagnosis of corneal and conjunctival pathologies, with a focus on malignant lesions. In this pilot study on 82 lesions, they concluded that HR-OCT was helpful to determine the etiology and to differentiate between multiple ocular surface lesions, including ocular surface squamous neoplasia, pterygium, nevi, and melanoma, as well as to evaluate resolution after treatment. However, this imaging technique was less useful in evaluating pigmented lesions. Even though this imaging technique cannot replace either clinical evaluation or histopathologic diagnosis, it can be an important aid in determining the diagnosis of ocular surface pathology and in determining disease resolution [24].

### 3.3. UBM and AS-OCT

Buchwald et al. studied 13 conjunctival and 25 eyelid lesions. The authors concluded that, in solid tumors, the final diagnosis cannot be made based on UBM or AS-OCT alone [1]. In general, when using AS-OCT it was impossible or uncertain to determine the tumor depth. When comparing AS-OCT and UBM, AS-OCT is the better imaging technique for small cystic structures. This is especially useful for nevi as they often contain small cysts. In contrast, UBM is a better technique to determine the tumor margins [1].

Bianciotto et al. studied 200 eyes with anterior segment tumors. Even though they only included 6 conjunctival tumors, their study resulted in interesting conclusions. Comparison of UBM and AS-OCT showed that UBM had a better tumor visualization and better resolution of the posterior margin. UBM also had a better resolution for pigmented as well as for nonpigmented tumors. However, AS-OCT had better resolution of the anterior border and better resolution of the anterior segment anatomy. Posterior tumor shadowing was rarely found in UBM images and more common in AS-OCT. The image quality was good in UBM but less in AS-OCT. This study shows that AS-OCT is superior to UBM for the imaging of conjunctival lesions, because AS-OCT offers a higher resolution and conjunctival lesions are superficial and mostly not pigmented. Their findings demonstrate that AS-OCT is affected by optical shadowing in large pigmented lesions. AS-OCT uses light, which is more comfortable for the patient, but the light is blocked by opaque tissues which results in lower penetration depth. This is a crucial factor in the evaluation and treatment of anterior segment tumors. They conclude that AS-OCT is a useful tool in the evaluation of superficial nonpigmented lesions of the eye, although AS-OCT suffers from poor resolution and shadowing in large or pigmented lesions. In comparison, UBM can penetrate better through the lesion, which results in better visualization of the posterior tumor border and whole tumor configuration [25].

## 4. Discussion 

These studies showed that AS-OCT and UBM both have their advantages and disadvantages because of their specific characteristics. None can replace histopathological examination for diagnosis but they both give useful information helping in the differential diagnosis [1, 19]. Both noninvasive imaging techniques provide useful information about the thickness and internal characteristics and extension in depth and size and shape of conjunctival and corneal tumors.

Although AS-OCT technically has a higher resolution than UBM [9, 10], UBM seems to be superior in accuracy of tumor visualization, quality of the image, resolution of the posterior margin, and resolution of pigmented and nonpigmented tumors [1, 24, 25]. UBM is also able to visualize all margins more often than AS-OCT [1, 25]. Therefore UBM is a useful tool in delineating the margins and measuring the extent of a conjunctival lesion before surgical excision [20]. Another advantage of UBM is that tumor shadowing is rarely seen, while this is much more frequently seen on AS-OCT [8, 9, 24, 25]. For this reason, AS-OCT is not preferable for the visualization of large pigmented lesions [25]. In contrast, AS-OCT provides a better resolution of the anterior margin and anterior segment anatomy [25].

For thicker lesions as a* limbal dermoid*, UBM is the preferred technique. UBM has proven to be valuable in measuring the depth and extension of dermoids [17, 18] and also in establishing a differential diagnosis [17]. Since UBM can accurately measure depth of invasion, it is therefore very useful in the preoperative evaluation of a limbal dermoid [16, 18].

*Conjunctival nevi* often contain typical small cysts. Lin et al. demonstrated that UBM is useful in visualizing these cysts in pigmented conjunctival lesions [20]. Shields et al. and Buchwald et al. found that AS-OCT can identify these small cystic structures more accurately than UBM, and, therefore, AS-OCT is a useful tool in investigating these lesions [1, 22]. AS-OCT also has a high correlation with clinical examination and histopathology in visualizing these intrinsic cysts [22]. It was also found that AS-OCT can visualize all margins of conjunctival nevi, even the deep margins, although deep optical shadowing is often seen, especially in pigmented nevi [22]. For conjunctival nevi we can conclude that AS-OCT seems to be more accurate in assessing the extent of these tumors as long as the nevus is not very thick and not heavily pigmented.

Ho et al. investigated* conjunctival melanomas* and found that UBM is a very good technique for determining the posterior margin and estimating tumor thickness. Therefore, UBM is useful in determining the excision depth important in the planning of a surgical resection [21]. AS-OCT was not compared to UBM in conjunctival melanomas.

Concerning* squamous cell carcinoma (SCC)*, HR-OCT was found to be useful in the differentiation between SCC and similar lesions like amelanotic melanoma and corneal fibrosis [10, 24]. Studies which concentrated on the differentiation between SCC and pterygia concluded that AS-OCT is a good diagnostic tool to differentiate SCC from pterygia [8, 10, 24]. AS-OCT can differentiate between these two lesions by measuring the difference in epithelial thickness [8]. Furthermore, the authors found that, for SCC as well as pterygia, there is a good correlation between the findings on UHR-OCT and histopathology [8]. Also Nanji et al. found that for SCC HR-OCT correlated well with histopathology [24]. HR-OCT can also be used for the monitoring of the resolution of SCC during therapy. In this way, HR-OCT can detect subtle residual epithelial thickening which is not visible on clinical examination. This prevents premature termination of treatment.

Shousha et al. found that UHR-OCT is a good technique when visualizing* conjunctival and corneal intraepithelial neoplasia (CCIN)*. UHR-OCT is particularly useful for the confirmation of recovery of CCIN. This is important because UHR-OCT could in this way replace a biopsy, which is harmful to the surface of the eye and which can be false negative because of sampling error. Disadvantages of UHR-OCT concerning CCIN are the fact that microinvasion cannot be excluded and the fact that the resolution of UHR-OCT is not high enough to assess intracellular characteristics [9].

UBM and AS-OCT both have a strong correlation with histopathology and they can both assess the structure and the extent of lesions in order to guide treatment [8, 10, 24]. When tumor invasion of the sclera or cornea is documented before resection, the surgeon can prepare and counsel the patient for a more extensive resection with graft or even discuss the possibility of an enucleation or exenteration.

A major limitation of most studies was the small study population, often leading to the conclusion that further research is needed. Furthermore, only a limited amount of tumor types was investigated, which makes it impossible to extrapolate these findings to all corneal and conjunctival tumors.

## 5. Conclusion

The literature shows that AS-OCT and UBM are both very useful and complementary techniques for the evaluation and follow-up of corneal and conjunctival tumors even though they cannot replace histopathological analysis for the diagnosis. Due to their different measuring technique, they have different advantages and disadvantages. The disadvantage of AS-OCT is that it cannot penetrate deeper than 1–3 mm and cannot penetrate through pigmented lesions. But for smaller lesions AS-OCT is a more accurate technique that can give detailed images of the remaining healthy cornea, can identify cysts, or might be useful in detecting tumor recurrence. For larger or pigmented lesions UBM can better delineate tumor margins and tumor thickness. More comparative studies are needed to investigate which imaging technique is most suitable for a certain tumor type.

## Figures and Tables

**Table 1:** Articles using UBM in conjunctival and corneal tumors.

Author, year	Study	Number of patients	Imaging technique	Aim	Tumor type	Results	Conclusion
*UBM*							

Lanzl et al., 1998	The role of ultrasound biomicroscopy in surgical planning for limbal dermoids—study design not given	2	UBM (50 MHz)	To investigate the use of ultrasound biomicroscopy (UBM) examination for surgical planning in limbal dermoids	Infantile limbal dermoid	*UBM* (i) Can distinguish normal cornea from the more sonolucent lesion (ii) Presence or absence of Descemet's membrane could be identified (iii) Depth of involvement of limbal dermoids could be assessed	*UBM* (i) Can assess depth involvement of opaque corneal lesions such as limbal dermoids (ii) Because planning of the surgical approach in these cases is facilitated by preoperative knowledge about the depth of penetration of these opaque lesions, UBM can be regarded as a helpful tool in the clinical management

Grant and Azar, 1999	Ultrasound biomicroscopy in the diagnosis and management of limbal dermoid—study design not given	1	UBM (type of probe not mentioned)	To report the use of ultrasound biomicroscopy in the diagnosis and management of limbal dermoid	Limbal dermoid	Clinical examination, ultrasound biomicroscopy, biopsy confirmed the diagnosis of limbal dermoid (highly echogenic lesion, 0.78 mm thick)	*UBM* (i) Useful diagnostic adjunct for limbal dermoids (ii) Helpful in delineating the extent of these lesions

Hoops et al., 2001	Preoperative evaluation of limbal dermoids using high resolution biomicroscopy—retrospective analysis	8	High resolution biomicroscopy (50 MHz)	To assess whether ultrasound biomicroscopy (UBM) can detect the corneal depth of penetration of dermoids which could improve planning of surgery	Limbal dermoids	(i) 7/8: UBM showed a more reflective and predominantly homogeneous lesion compared with the unaffected corneal stroma, so that the lateral margins of the lesion could be clearly identified (ii) Penetration of the tumor: 4/8: incomplete stromal penetration of the dermoid was noticed; 1/8 showed a corneal full-thickness dermoid; 1/8 intraocular protrusion; 2/8 remained unclear because of reduced compliance (iii) 7/8: Descemet's membrane beneath the dermoid could not be visualized because of strong sound attenuation inside the lesion	*UBM* (i) Improves the preoperative evaluation of limbal dermoids (ii) Subtle examination technique for the depth of corneal penetration is required because of the strong sound attenuation in this tissue, reducing the visibility of deep corneal structures

Buchwald et al., 2003	Ultrasound biomicroscopy of conjunctival lesions—prospective study	28	UBM (30 MHz)	To determine the value of UBM in the diagnosis of conjunctival lesions	28 conjunctival lesions: Compound nevus (8/28), cysts (6/28), inflammatory processes (3/28), granulomatous processes (2/28), lymphomas (2/28), foreign bodies (2/28), pterygium (2/28), malignant melanoma (1/28), primary acquired melanosis (1/28), conjunctival amyloidosis (1/28)	*UBM* (i) Cyst of conjunctiva: demonstrating cystic tumor in 21% (ii) Solid tumor of conjunctiva: no definite diagnosis with UBM (iii) Compound nevus: heterogeneous sonographic structure within the tumor (iv) Foreign body: posterior shadowing of the underlying tissue	*UBM* (of conjunctival lesions caused by a cyst or a solid tumor) (i) May be an additional diagnostic tool (ii) Now not possible to differentiate between different lesions solely by means of ultrasonography

Lin et al., 2004	Ultrasound Biomicroscopy in Pigmented Conjunctival Cystic Nevi—study design not given	2	UBM (type of probe not mentioned)	To report the use of UBM in the clinical diagnosis and management of pigmented conjunctival cystic nevi	2 conjunctival lesions: raised melanocytic lesions localized on the conjunctiva characterised by rapid growth; they were suspected to be inflamed juvenile conjunctival nevi	*UBM* (i) Multiple areas of cystic tissue, which is compatible with pathologic finding of compound nevus with epithelial inclusion cysts formation (ii) Clear interface between the mass and the underlying sclera was found	*UBM* (i) Useful diagnostic to distinguish cysts in conjunctival lesions (ii) May be helpful in delineating the extent of lesions prior to excision

Ho et al., 2007	Ultrasound biomicroscopy for estimation of tumor thickness for conjunctival melanoma—retrospective review	3	UBM (50 MHz)	To assess the feasibility of using high frequency UBM in estimating thickness of conjunctival melanomas preoperatively	Conjunctival melanoma (3)	Patient 1: UBM thickness = 1.99 mm and Breslow thickness = 1.5 mm Patient 2: UBM thickness = 2.4 mm and Breslow thickness = 2.23 mm Patient 3: both UBM and Breslow thickness = 2.3 mm	*UBM* (i) Useful tool for estimation of tumor thickness for conjunctival melanomas (ii) Additional diagnostic tool for estimating tumor thickness before surgical resection of conjunctival melanoma

**Table 2:** Articles using AS-OCT in conjunctival and corneal tumors.

Author, year	Study	Number of patients	Imaging technique	Aim	Tumor type	Results	Conclusion
*AS-OCT*							

Shields et al., 2011	Anterior segment optical coherence tomography of conjunctival nevus—retrospective interventional case series	22 eyes of 21 patients	AS-OCT (1310 nm)	To evaluate conjunctival nevi using AS-OCT	Conjunctival nevi (22)	*AS-OCT* (i) All margins of the nevus, including the deep margin, could be visualized with high resolution of the anterior margin in 100% of cases, posterior margin in 82% of cases, lateral margin in 86% of cases (ii) Intrinsic cysts within the nevus were detected in 17 cases (77%)	*AS-OCT* (i) Provides high resolution imaging of conjunctival nevi (ii) Can demonstrate all margins (iii) Can provide information on the presence of intralesional cysts, for diagnosis (iv) Main drawback: optical shadowing of deeper structures from pigment within nevi

Welch et al., 2011	Pterygia measurements are more accurate with anterior segment optical coherence tomography (a pilot study)—study design not given	13	AS-OCT (wavelength not mentioned)	To compare standard slit-lamp beam measurements of pterygia to computer caliper measurements of AS-OCT images	Pterygia	*Slit lamp versus OCT* (i) Slit lamp: the mean of the differences was 0.3 and the standard deviation was 0.32 (ii) OCT: the mean of the differences was 0.1 and the standard deviation was 0.12. A two-tailed *t*-test demonstrated a statistically significant difference in these measurements (*p* = 0.0256)	*AS-OCT* (i) Significantly more reproducible results than the slit-lamp beam for measurements of the distance of a pterygium's apex from the limbus (ii) May provide more accurate clinical assessment of extension of pterygia onto the cornea and may be useful for research purposes

Shousha et al., 2011	Diagnosis and management of conjunctival and corneal intraepithelial neoplasia using ultrahigh resolution optical coherence tomography—prospective, noncomparative, interventional case series	7	UHR-OCT (870 nm)	To report a novel diagnostic technique and a case series of CCIN diagnosed and followed up using prototype UHR-OCT	Conjunctival and corneal intraepithelial neoplasia (CCIN)	*UHR-OCT* (i) Thickened hyperreflective epithelium and abrupt transition from normal to hyperreflective epithelium in all 7 cases (ii) Excellent correlation with histopathologic specimens	*UHR-OCT* (i) Useful to diagnose and manage medically treated CCIN (ii) Could prevent the premature termination of topical treatment in the presence of subclinical disease (iii) A larger sample size is needed for further validation of its sensitivity and specificity

Kieval et al., 2012	Ultrahigh resolution optical coherence tomography for differentiation of ocular surface squamous neoplasia (OSSN) and pterygia—prospective case series	34 eyes of 34 patients	UHR-OCT (840 nm)	To assess the use of an UHR-OCT as an adjuvant diagnostic tool in distinguishing OSSN and pterygia	Conjunctival lesions (34) clinically suspicious for OSSN or pterygia	*UHR-OCT* (for differentiating between OSSN and pterygia) (i) Sensitivity 94% (ii) Specificity 100% (cut-off value of 142 *μ*m)	*UHR-OCT* (for OSSN and pterygia) (i) Statistically significant difference in epithelial thickness (ii) Significant degree of morphologic correlation with the histopathologic results

Shousha et al., 2013	Diagnosis of ocular surface lesions using ultrahigh resolution optical coherence tomography—prospective, noncomparative, interventional case series	54	UHR-OCT (840 nm)	To assess the use of ultrahigh resolution (UHR) optical coherence tomography (OCT) in the diagnosis of ocular surface lesions	24 conjunctival lesions, 19 corneoconjunctival lesions, 11 corneal lesions: primary acquired melanosis lesions (8/54), amelanotic melanoma lesions (5/54), nevi (2/54), ocular surface squamous neoplasia lesions (19/54), histiocytosis lesion (1/54), conjunctival lymphoma lesions (6/54), conjunctival amyloidosis lesions (2/54), pterygia lesions (11/54)	*UHR-OCT* (i) Close correlation with the obtained histopathologic specimens (ii) When clinical differential diagnosis of ocular surface lesions was broad, UHR-OCT images provided optical signs indicating a more specific diagnosis and management (iii) Amelanotic melanoma, conjunctival amyloidosis, primary histiocytosis and, in 1 case of OSSN, UHR-OCT was instrumental in guiding the diagnosis. In those cases, UHR-OCT suggested that the presumed clinical diagnosis was incorrect and favored a diagnosis that later was confirmed by histopathologic examination	*UHR-OCT* (i) Correlations between UHR-OCT and histopathologic findings confirm that UHR-OCT is an adjunctive diagnostic method that can provide a noninvasive means to help guide diagnosis and management of ocular surface lesions

Nanji et al., 2015	High resolution optical coherence tomography as an adjunctive tool in the diagnosis of corneal and conjunctival pathology—prospective case series	82	HR-OCT (830 nm)	To evaluate the use of a commercially available, high resolution, spectral-domain optical coherence tomography (HR-OCT) device in the diagnosis of corneal and conjunctival pathologies, with a focus on malignant lesions	Location of lesions not mentioned: Normal eyes (10), OSSN (21), pterygium or pingueculum (24), lymphoma (3), pigmented conjunctival lesions (nevus, flat melanosis, or melanoma) (18) and Salzmann nodular degeneration (6)	*HR-OCT* (i) Useful in differentiating among various lesions based on optical signs (ii) OSSN: epithelial thickening and hyperreflectivity (iii) Pterygia and pinguecula: subepithelial mass under thinner epithelium (iv) Lymphoma: hyporeflective, homogenous subepithelial mass (v) Pigmented lesions: differentiation was more difficult, but certain characteristics could be identified. Eyes with nevi and melanoma displayed both intensely hyperreflective basal epithelial layers and discrete subepithelial lesions but could be differentiated by the presence of cysts in nevi and intense shadowing of sublesional tissue in most melanomas	*HR-OCT* (i) Useful noninvasive adjunctive tool in the diagnosis of ocular surface lesions

**Table 3:** Articles describing the use of UBM and AS-OCT in conjunctival and corneal tumors.

Author, year	Study	Number of patients	Imaging technique	Aim	Tumor type	Results	Conclusion
*UBM versus AS-OCT*							

Buchwald et al., 2003	Optical coherence tomography versus ultrasound biomicroscopy of conjunctival and eyelid lesions—prospective study	38 tumors of 35 patients	UBM (30 MHz) and AS-OCT (1310 nm)	To compare the value of UBM and AS-OCT in the diagnosis of conjunctival and eyelid lesions	13 conjunctival lesions and 25 eyelid lesions: pterygium (8/38), seborrheic keratosis (7/38), cyst of the eyelid (5/38), basal cell carcinoma of the eyelid (4/38), compound nevus of the conjunctiva (4/38), chalazion (3/38), primary acquired melanosis (1/38), actinic keratosis (1/38), nevus (1/38), cavernous haemangioma (1/38), melanoma in situ (1/38), foreign body (1/38), epidermoid cyst (1/38)	*AS-OCT* (i) More reliable imaging: small cystic structures of compound nevus (ii) Assessment of the margins of the tumors (particularly in depth) was impossible or uncertain *AS-OCT and UBM* Solid tumors: the definite diagnosis could not be differentiated by UBM or OCT alone	*OCT* Show very small cystic structures more distinctly than UBM *UBM* Better for assessing the margins of the tumor than OCT

Bianciotto et al., 2011	Assessment of anterior segment tumors with ultrasound biomicroscopy versus anterior segment optical coherence tomography in 200 cases—retrospective, noninterventional case series	200	UBM (50 MHz probe) and AS-OCT (1310 nm)	To compare UBM versus AS-OCT for imaging of tumors of the anterior segment of the eye	6 conjunctival lesions (diagnosis not mentioned), 0 corneal lesions, 194 other locations: nevus (75/200), melanoma (47/200), cyst (48/200), epithelioma (adenoma) (5/200), metastasis (4/200), melanocytosis (4/200) and melanocytoma (4/200), others (3/200)	*UBM* (i) Better overall tumor visualization (138 [69%] versus 62 [31%]) (ii) Better resolution of the posterior margin (147 [74%] versus 53 [27%]) (iii) Better resolution for pigmented tumors (*n* = 162; 107 [66%] versus 55 [34%]) (iv) Better resolution for nonpigmented tumors (*n* = 38; 23 [61%] versus 15 [39%]) *AS-OCT* (i) Better resolution of the anterior margin (40 [20%] versus 160 [80%]) (ii) Better overall resolution of anterior segment anatomy (41 [21%] versus 159 [80%])	*UBM > AS-OCT* (i) Anterior segment tumors: better visualization of the posterior margin and overall better images for entire tumor configuration
